# Air pollution and urinary n-acetyl-B-glucosaminidase levels in residents living near a cement plant

**DOI:** 10.1186/s40557-016-0138-8

**Published:** 2016-09-29

**Authors:** Min Soo Jung, Jae Yoon Kim, Hyun Seung Lee, Chul Gab Lee, Han Soo Song

**Affiliations:** 1 Department of Occupational & Environmental Medicine, School of Medicine, Chosun University, 309 Pilmun-daero Dong-gu, Gwangju, 61452 South Korea; 2 Korea Medical Institute, 5-6, Sangmujungang-ro 78beon-gil, Seo-gu, Gwangju, 61949 South Korea

**Keywords:** Cement, Heavy metal, NAG, Air pollution, Mercury, Cadmium

## Abstract

**Background:**

To identify adverse renal effects due to air pollution derived from a cement plant in Korea. Urinary n-acetyl-B-glucosaminidase (U-NAG) levels in residents living near a cement plant were compared to those in a group who lived farther away from the plant.

**Methods:**

From June to August 2013 and from August to November 2014, laboratory tests for U-NAG and heavy metal were conducted on 547 study participants. Based on the level of air pollution exposure, subjects were divided into the “less exposed group,” (LEG) which consisted of 66 persons who lived more than 5 km away from the cement plant, the “more exposed group from the rural area” (MEG-R), which consisted of 272 persons, and the “more exposed group from downtown area” (MEG-D), which consisted of 209 persons who lived within a 1 km radius of the cement plant. U-NAG levels >5.67 U/L were defined as “higher U-NAG” levels. We compared the prevalence of higher U-NAG levels and estimated the adjusted odds ratio (OR) by air pollution exposure using a chi-square test and multiple logistic regression analysis. Further, we estimated the interaction between air pollution exposure and heavy metal exposure in renal toxicity.

**Results:**

The OR of higher U-NAG levels by MEG-D and MEG-R compared to LEG was 2.13 (95 % CI 0.86–4.96) and 4.79 (95 CI 1.65–10.01), respectively. Urinary cadmium (U-Cd), urinary mercury (U-Hg), age, occupation, hypertension, and diabetes had a significant association with higher U-NAG levels. However, blood lead (B-Pb), sex, and smoking were not associated with higher U-NAG. Especially, concurrent exposure to heavy metals (U-Hg or/and U-Cd) and air pollution had an additive adverse effect. In the group with both 4^th^ quartile heavy metal exposure (U-Cd or/and U-Hg) and air pollution exposure, the OR in MEG-R and MEG-D was 6.49 (95 % 1.42–29.65) and 8.12 (95 % CI 1.74–37.92), respectively, after adjustment for age, occupation, hypertension, diabetes.

**Conclusions:**

U-NAG levels seem to be affected by air pollution exposure as well as age, hypertension, diabetes, and even low levels of cadmium and low levels of mercury. Moreover, concurrent exposure to heavy metals and air pollution can have additive cytotoxic renal effects.

## Background

Many studies definitively show that air pollution contributes to premature death [1, 2], cardiovascular disease [3, 4], and respiratory disease such as asthma and chronic obstructive pulmonary disease [3, 5, 6], based on both the short-term and long-term effects of exposure. Individuals for whom air pollution poses a particular health risk include fetuses, children, and older people, especially those in low-income societies or in a polluted environment [7–9].

**Table 1:** General characteristics of study population by air pollution

Variables	Total		LEG		MEG-R		MEG-D		*P*-value
	*n*	%	*n*	%	*n*	%	*n*	%	
Sex									
Male	203	37.1	19	28.8	105	38.6	79	37.8	0.323
female	344	62.9	47	71.2	167	61.4	130	62.2	
Age, years									
40–59	185	33.8	19	28.8	96	35.3	70	33.5	0.600
≥ 60	362	66.2	47	71.2	176	64.7	139	66.5	
Occupation									
non-manual	370	67.6	28	42.4	162	59.6	180	86.1	<0.001
manual	177	32.4	38	57.6	110	40.4	29	13.9	
Smoking									
none	452	82.6	57	86.4	226	83.1	169	80.9	0.567
current	95	17.4	9	13.6	46	16.9	40	19.1	
HTN, mmHg									
< 140/90	410	75.0	50	75.8	208	76.5	152	72.7	0.635
≥ 140/90	137	25.0	16	24.2	64	23.5	57	27.3	
HbA1c, %									
< 6.5	449	82.1	46	69.7	223	82.0	180	86.1	0.010
≥ 6.5	98	17.9	20	30.3	49	18.0	29	13.9	
Total	547	100.0	66	100.0	272	100.0	209	100.0	

*p*-value by Chi-square test; non-manual: office work; the unemployed; manual: farmer, labor, construction, blue collar job

*LEG* ‘less exposed group’ who lived 5 km or more away from the cement plant

*MEG* ‘more exposed group’ who lived within the 1 km radius of the cement plant

*MEG-R* MEG in rural area; MEG-D: MEG in downtown

**Table 2:** Geometric mean of U-NAG and prevalence of higher U-NAG by related factors

Variables	U-NAG, U/L		P*	Higher U-NAG (≥5.67), U/L		P**
	GM	GSD		*n*	%	
Sex						
Male	1.90	1.49	0.004	35	17.2	0.752
Female	2.51	1.79		63	18.3	
Age, years						
40–59	1.71	1.05	0.000	22	11.9	0.009
≥ 60	2.62	1.80		76	21.0	
Occupation						
non-manual	2.14	1.66	0.070	62	16.8	0.307
manual	2.55	1.62		36	20.3	
Air pollution exposure						
LEG	2.63	1.35	0.242	8	12.1	0.071
MEG-R	2.12	1.45		43	15.8	
MEG-D	2.36	2.34		47	22.5	
Smoking						
none	2.30	1.82	0.448	83	18.4	0.552
current	2.10	1.65		15	15.8	
HTN, mmHg						
< 140/90	2.17	1.59	0.101	66	16.1	0.055
≥ 140/90	2.57	2.48		32	23.4	
DM, HbA1c (%)						
< 6.5	2.14	1.52	0.007	71	15.8	0.006
≥ 6.5	2.93	3.55		27	27.6	
Urine cadmium, μg/g cr						
1Q (0.09–0.44)	1.93	0.90	0.007	17	12.6	0.145
2Q (0.44–0.75)	1.99	1.69		24	17.4	
3Q (0.75–1.16)	2.40	2.38		25	18.2	
4Q (1.16–8.91)	2.84	2.43		32	23.4	
Urine mercury, μg/g cr						
1Q (0.09–0.42)	1.96	1.05	0.020	19	14.1	0.023
2Q (0.42–0.63)	1.98	1.33		19	13.8	
3Q (0.63–0.93)	2.56	1.50		24	17.5	
4Q (0.93–4.16)	2.65	3.48		36	26.3	
Blood lead, μg/dL						
1Q (0.77–2.13)	2.33	1.66	0.941	25	18.8	0.830
2Q (2.13–2.70)	2.31	1.74		27	19.3	
3Q (2.70–3.50)	2.17	1.91		25	18.2	
4Q (3.50–10.37)	2.26	1.72		21	15.3	
Total	2.27	1.80		98	17.9	

*p-value by independent *t*-test, ANOVA, calculated by logarithmically transforming variables

**p-value by Chi-square test

**Table 3:** OR of U-NAG by related factors and air pollution exposure

Variables	Unadjusted			Adjusted		
	OR	95 % CI		OR	95 % CI	
Sex						
male						
female	1.07	0.68	1.69	0.73	0.39	1.37
Age, year						
40–59						
≥ 60	1.96	1.18	3.28	1.81	1.05	3.13
Occupation						
non-manual						
manual	1.26	0.80	2.00	1.78	1.03	3.07
Smoking						
none						
current	0.83	0.45	1.52	0.88	0.43	1.82
Air pollution exposure						
LEG						
MEG-R	1.36	0.60	3.05	2.07	0.86	4.96
MEG-D	2.10	0.93	4.71	4.07	1.65	10.01
Hypertension, mmHg						
< 140/90						
≥ 140/90	1.58	0.98	2.55	1.67	1.00	2.78
DM, HbA1c (%)						
< 6.5						
≥ 6.5	2.02	1.21	3.37	2.51	1.43	4.40
Urine cadmium, μg/g cr						
1Q (0.09 ~ 0.44)						
2Q (0.44 ~ 0.75)	1.46	0.74	2.86	1.46	0.71	3.02
3Q (0.75 ~ 1.16)	1.54	0.79	3.02	1.51	0.72	3.13
4Q (1.16 ~ 8.91)	2.11	1.11	4.02	2.20	1.03	4.67
Urine mercury, μg/g cr						
1Q (0.09 ~ 0.42)						
2Q (0.42 ~ 0.63)	0.97	0.49	1.93	0.96	0.46	2.01
3Q (0.63 ~ 0.93)	1.29	0.67	2.49	1.30	0.64	2.63
4Q (0.93 ~ 4.16)	2.17	1.17	4.03	2.65	1.34	5.25
Blood lead, μg/dL						
1Q (0.77 ~ 2.13)						
2Q (2.13 ~ 2.70)	1.03	0.56	1.88	0.96	0.49	1.87
3Q (2.70 ~ 3.50)	0.96	0.52	1.78	0.89	0.44	1.77
4Q (3.50 ~ 10.37)	0.78	0.41	1.47	0.67	0.32	1.41

Logistic regression with adjusted for all variables; *CI* confidence interval

**Table 4:** OR of higher U-NAG by heavy metals and air pollution exposure

Variables				Unadjusted			Adjusted^a^		
	higher heavy metals	Air pollution	*n*	OR	95 % CI		OR	95 % CI	
Model 1	None		320	1			1		
	U-Hg or U-Cd		180	1.79	1.11	2.87	2.03	1.22	3.36
	U-Hg and U-Cd		47	2.66	1.32	5.36	3.32	1.55	7.11
Model 2	None	LEG	36	1			1		
		MEG-R	153	1.98	0.43	9.05	2.13	0.45	9.95
		MEG-D	131	4.21	0.94	18.66	4.79	1.05	21.80
	U-Hg or/and U-Cd	LEG	30	4.25	0.78	22.88	4.33	0.78	23.89
		MEG-R	119	4.98	1.12	22.12	6.49	1.42	29.65
		MEG-D	78	6.26	1.38	28.38	8.12	1.74	37.92

Model 1: Concurrent exposure of heavy metals; Model 2: Interaction between air pollution and heavy metal

Logistic regression method

^a^Adjusted by sex, age, HTN, DM

Airborne particulate matter (PM) as a main composition of air pollution has various cytotoxic effects according to source, physical properties, and chemical speciation [10]. PM could influence the activation of inflammatory cytokines, oxidative stress, and cytotoxic reaction by systemic absorption [11]. Therefore, it not only affects the direct contact site such as the respiratory system, but can also have other effects, such as on the cardiovascular system or the nervous system [12–14], or on diabetes mellitus [15, 16].

The health effects of air pollution on cardiovascular diseases and respiratory disease have attained national and international attention, whereas the renal effects seem to be neglected. However, studies on the general health effects of air pollution indicate increased renal disease [17]. Diesel exhaust triggered renal toxicity in an animal experimental study. Thus we explored the relationship between air pollution and renal effects. There is some evidence that second-hand smoke causes adverse renal effects in children with chronic renal disease [18, 19]. Current cigarette smoking is a risk factor for chronic kidney disease [20]. A longitudinal analysis using a validated spatiotemporal model showed that long-term PM2.5 exposure negatively affects renal function [17]. However, little is known about the effects of air pollution on renal disease.

Major risk factors of chronic kidney disease are age, diabetes mellitus, and hypertension. However, unknown risk factors also play an important role. Lower level exposure to environmental nephrotoxicants, such as heavy metals, in the general population has been shown to have a significant adverse effect on kidney disease [21–24]. Some articles show that environmental exposure to heavy metals is associated with a decreased glomerular flow rate (GFR) or high prevalence of chronic kidney disease [25, 26]. There are a few articles suggesting the probability of a relationship between chronic kidney disease and diesel engine exhaust [27], passive smoking, and fine particulate matter [27].

In 2013, the authors of the current study investigated the health effects of air pollution on a population living near a cement plant. In that study, we reported the effects on respiratory disease of cement air pollution [28, 29]. The aim of this article is to identify the relationship between chronic kidney disease and air pollution from a cement plant, using urinary n-acetyl-B-glucosaminidase (U-NAG) as a biomarker for damage to the renal proximal convoluted tubule [30].

## Methods

### Participants

The participants in this study were local residents who took part in “The survey of health effects for residents around a cement plant and mines in Jangseong-gun” conducted in 2013 [29]. From June to September 2013, this epidemiological study, which was requested by the National Institute of Environmental Research, was conducted to evaluate the long term health effects of particulate dust exposure on residents who lived near a cement plant located in Jangseong-gun, South Korea. To investigate the impact of air pollution, the residents who lived within a 1 km radius of the cement plant were designated as the “more exposed group” (MEG), and those who lived more than 5 km away from the cement plant were assigned to the “less exposed group” (LEG). The LEG served as a control group [29]. Further, we divided MEG into “more exposed group in rural area” (MEG-R) and “more exposed group in downtown area” (MEG-D), because of differences by demographic configuration. According to a 2013 air pollution measurement, the average PM10 concentration was 38.52 microgram per cubic meter (μg/m^3^) in the LEG area and 43.27 ~ 45.53 μg/m^3^ in the MEG area. Average PM2.5 concentration in the atmosphere was 19.34 μg/m^3^ in the LEG area and 24.44 ~ 25.48 μg/m^3^ in the MEG area. There was a significant difference (both PM10 and PM2.5) between MEG-R and MEG-D. Average cadmium concentration in the atmosphere was 1.2 ng/m^3^ in the LEG area and 1.4 ~ 1.6 ng/m^3^ in the MEG area. Average lead concentration in the atmosphere was 21.7 ng/m^3^ in the LEG area and 38.6 ~ 47.4 ng/m^3^ in the MEG area. Average SiO_2_concentration in the atmosphere was 0.384 μg/m^3^ in the LEG area and 0.445 ~ 0.739 ng/m^3^ in the MEG area. A stratified sampling method was used to select 600 samples out of 1375 residents over age 40 who lived around the cement plant. These individuals were stratified according to sex, age, and residential area. In the current study, to evaluate the health effects of heavy metals, laboratory tests for determining urinary n-acetyl-B-glucosaminidase (U-NAG) and heavy metal were conducted. After the purpose, content, and a detailed plan of the sample survey was explained to the subjects, those who gave consent participated in the procedure. The final sample size was 547 individuals: 66 from LEG, 272 from MEG-R, and 209 from MEG-D. The Institutional Review Board (IRB) of the National Institute of Environmental Research provided ethical approval for the study.

### Laboratory analysis methods

Subjects were interviewed by a clinician prior to the test, and each subject’s information such as age, area of residence, current job, and smoking habits were obtained through a questionnaire. Blood tests and urine tests were performed and analyzed to determine levels of blood lead (B-Pb), urinary cadmium (U-Cd), urinary mercury (U-Hg), hemoglobin A1c (HbA1c), and U-NAG. An EDTA tube was used to collect 3 ml of whole blood, which was mixed by roller mixer to analyze B-Pb. Spot urine was collected and moved into each polypropylene tube and mixed by roller mixer in about 20 min to analyze U-Cd and U-Hg. Urine samples for elemental analysis were stored at below 4 °C (−8 ~ 2 °C) until analysis. B-Pb was measured by autosampler (Varian atomic absorption spectrophotometer, Varian SpectrAA-880U) using the flameless method and analyzed by Varian SpectrAA-880U. Urinary Hg was measured by automatic mercury analyzer (SP-3DS, NIC) using the gold-amalgam method. U-Cd levels were measured by the autosampler and Atomic absorption spectrophotometer, Perkin-Elmer Model AAnalyst 600 Zeeman Correction using the flameless method. U-NAG levels were measured by colorimetry (kit from Shionogi, Japan). U-NAG levels were adjusted with creatinine into U-NAG/Cr (U/g cr). To evaluate the reliability of the laboratory test for heavy metals, we requested results from two laboratory institutions. The intraclass correlation coefficient (ICC) was analyzed for 10.6 % (58 cases) of the total samples. ICC of B-Pb, U-Cd, and U-Hg were 0.923, 0.841, and 0.874, respectively.

### Variables

#### Independent variables

Factors related to general characteristics (e.g., sex, age, occupation, smoking, hypertension, diabetes mellitus) were classified as independent variables. Age was divided into the following groups: 40–59 and ≥60 years. Occupation was categorized into the following groups: non-manual work and manual work (e.g., farmer, labor, construction, blue collar job). Based on the smoking status, subjects were classified as follows: non-smokers and current smokers. With respect to hypertension (HTN), subjects were divided into the following groups by measuring BP 2times: those with BP < 140/90 mmHg and those with BP ≥ 140/90 mmHg. With respect to diabetes mellitus (DM), subjects were divided into the following groups by measuring HbA1c levels: subjects with HbA1c <6.5 % and subjects with HbA1c ≥ 6.5 %. Air pollution exposure was categorized into the following groups: “less exposed group” (LEG), “more exposed group in rural area” (MEG-R), and “more exposed group in downtown area” (MEG-D). Heavy metal (B-Pb, U-Cd, U-Hg) were categorized into quartiles: 1^st^ quartile, 2^nd^ quartile, 3^rd^ quartile, and 4^th^ quartile.

#### Dependent variables

U-NAG was adjusted with creatinine into U-NAG/Cr (U/L). Higher U-NAG was defined as above 5.67 U/L, corresponding to 1 standard deviation on the basis of the standard values measured at analyzing institutions.

### Statistical analysis

The chi-square test was used to examine the relationship between higher U-NAG and sex, age, occupation, smoking, hypertension, and diabetes. U-NAG concentrations (the geometric mean and geometric standard deviation) were calculated by each variable. Unadjusted OR values were obtained using univariate logistic regression analysis to examine the correlation between higher U-NAG and each variable. Additionally, multivariate logistic regression analysis was carried out to adjust for all variables. Multivariate logistic regression analysis was conducted to examine the interaction between heavy metal and air pollution exposure after adjustment for sex, age, hypertension, and diabetes. Statistical significance was set to *p* < 0.05. All analyses were performed using SPSS v 21.0 (SPSS Inc., Chicago, IL, USA).

## Results

### General characteristics of the participants

The number of participants was 547. Mean age was 64.32(±11.02) years old. Among all participants, 37.1 % were male and 62.9 % were female. A total of 66.2 % were aged 60 or over. Of the LEG, 71.2 % were female, which was higher than the proportion of female subjects in MEG-R and MEG-D (61.4 and 62.2 %, respectively). This was not a significant difference. Of the LEG, 71.2 % were aged 60 or older, which was higher than the proportion in MEG-R and MEG-D (64.7 and 66.5 %, respectively). This was not a significant difference. Of the MEG-D, 19.1 % fell into the smoker category. This was higher than the proportions in MEG-R and LEG, which were 16.9 and 13.6 %, respectively. This was not a significant difference. Of the LEG, 30.3 % had diabetes, which was higher than the proportions in MEG-R and MEG-D, which were 18.0 and 13.9 %, respectively. As previously noted, LEG was older than MEG-R and MEG-D, and the percentage of women was higher in LEG than in MEG-R and MEG-D. The percentage of manual work was higher in LEG (57.6 %) than in MEG-R (40.4 %) and MEG-D (13.9 %) (Table 1).

### Prevalence of higher U-NAG by general characteristics and air pollution exposure

We calculated the geometric mean (GM) and geometric standard deviation (GSD) of U-NAG concentrations by each variable. GM and GSD of U-NAG concentrations from all participants were 2.27 U/L and 1.80 U/L, respectively. The percentage of “higher U-NAG” was 17.9 % (98 individuals). Sex, age, HbA1c, and U-Hg variables showed statistically significant differences in the geometric mean, or higher U-NAG. The GM of male and female U-NAG was 1.90 U/L (GSD 1.49) and 2.51 U/L (GSD 1.79), respectively. The ratio of higher U-NAG for the male category was 17.2 % and the ratio of higher U-NAG for the female category was 18.3 %. The GM of U-NAG of age younger than 60 was 1.71 U/L (GSD 1.05) and of age 60 or over was 2.62 U/L (GSD 1.80). This was a significant difference (*p* < 0.001). The ratio of higher U-NAG for people younger than 60 years old was 11.9 % and the ratio of higher U-NAG for people age 60 or older was 21.0 %. This was a significant difference (*p* = 0.009). The GM of U-NAG from non-DM was 2.14 U/L (GSD 1.52) and from DM was 2.93 U/L (GSD 3.55). This was a significant difference (*p* = 0.007). The ratio of higher U-NAG for non-DM was 15.8 % and the ratio of higher U-NAG for DM was 27.6 %. This was a significant difference. (*p* = 0.006). For U-Hg, as the quartile increased, the GM of U-NAG tended to increase. This was a significant difference (*p* = 0.020). For U-Hg, as the quartile increased, the ratio of higher U-NAG tended to increase. This was a significant difference. (*p* = 0.023) (Table 2).

### Higher U-NAG Related factors by multiple logistic regression analysis

Multivariate logistic regression analysis was conducted to examine the interaction between U-NAG and the variables. The OR values for each variable were adjusted for all variables. After variables were adjusted, age, occupation, air pollution, hypertension, diabetes, U-Cd, and U-Hg were found to have significant OR values. Sex, smoking, and B-Pb did not show significant OR values. The adjusted OR of the higher U-NAG in the old age group (≥60) was 1.81 (95 % CI 1.05 ~ 3.13) compared to the younger age group. The adjusted OR of the higher U-NAG in the manual work group was 1.78 (95 % CI 1.03 ~ 3.07) compared to the non-manual work group. The adjusted OR of the higher U-NAG in the MEG-R and MEG-D were 2.07 (95 % CI 0.86 ~ 4.96) and 4.07 (95 % CI 1.65 ~ 10.01), respectively compared to the LEG. The OR of the higher U-NAG in the HTN group was 1.67 (95 % CI 1.00 ~ 2.74) compared to the non-HTN group. The OR of the higher U-NAG in the DM group was 2.51 (95 % CI 1.43 ~ 4.40) compared to the non-DM group. The OR value increased as the quartile of U-Cd and U-Hg increased. As the concentration of U-Cd increased (2^nd^, 3^rd^, and 4^th^quartile), the OR value showed an increase: 1.46 (95 % CI 0.46 ~ 2.01), 1.51 (95 % CI 0.72 ~ 3.13), and 2.20 (95 % CI 1.03 ~ 4.67), respectively. As the concentration of U-Hg increased (2^nd^, 3^rd^, and 4^th^quartile), the OR value showed an increase: 0.96 (95 % CI 0.46 ~ 2.01), 1.30 (95 % CI 0.64 ~ 2.63), and 2.65 (95 % CI 1.34 ~ 5.25), respectively. In contrast, B-Pb did not show a significant association with higher U-NAG. (Table 3)

### OR of higher NAG by heavy metals and air pollution exposure

We performed a multiple logistic regression to evaluate the interaction between exposure to air pollution and heavy metals. We started by analyzing the effects of higher heavy metal levels, except for B-Pb, because B-Pb was not significantly associated with higher NAG.

The OR of U-NAG in the group with single exposure to higher heavy metal (U-Cd or U-Hg) and in the group with simultaneous exposure to higher heavy metals (U-Cd and U-Hg) were 2.03 (95 % CI 1.22 ~ 3.36) and 3.32 (95 % CI 1.55 ~ 7.11), respectively, compared to the group without heavy metals exposure (neither U-Cd nor U-Hg). Next, we stratified the analysis according to heavy metals exposure (U-Cd, U-Hg). In the group without heavy metals exposure (neither U-Cd nor U-Hg), the OR of MEG-R and MEG-D was 2.13 (95 % CI 0.45 ~ 9.95) and 4.79 (95 % CI 1.05 ~ 21.80), respectively, after adjustment. In the group with heavy metals exposure (U-Cd or/and U-Hg), the OR of the LEG, MEG-R, and MEG-D was 4.33 (95 % CI 0.78 ~ 23.89), 6.49 (95 % CI 1.42 ~ 29.65), and 8.12 (95 % CI 1.74 ~ 37.92), respectively, after adjustment by sex, age, HTN, and DM (Table 4).

## Discussion

Our study demonstrated the association between air pollution and urinary N-acetyl-beta-glucosaminidase (U-NAG). We used U-NAG as a biomarker for renal toxic damage in this research. U-NAG is a hydrolytic lysosomal enzyme in the proximal convoluted tubule [30]. U-NAG cannot pass through glomerular baseline membrane because it has 140,000 Dalton molecular weight. Thus U-NAG indicates proximal tubular damage at the cellular level. U-NAG level was significantly higher in patients with diabetes mellitus, microalbuminemic patients, and pregnant women with hypertensive disorders, compared to healthy controls [31]. In a study about biomarkers for renal dysfunction in type 2 Diabetes mellitus, U-NAG was the most sensitive marker of microalbuminuria and early renal damage, with sensitivity of 83.3 % compared to serum cystatin C, 2 renal tubular enzymes and neutrophil gelatinase associated lipocalin (NGAL), and β2-microglobulin (β2M) [32] [31]. U-NAG is a non-invasive and useful method of assessing renal tubular damage by environmental toxins [30].

We categorized heavy metal exposure (B-Pb, U-Cd, U-Hg) into quartiles: 1^st^ quartile, 2^nd^ quartile, 3^rd^ quartile, and 4^th^ quartile for estimating the dose-response relationship in order to analyze the relationship between U-NAG and heavy metal exposure. We found that the measured value of heavy metals did not correlate with the level of air pollution. Environmental exposure sources of heavy metals may be food, water, cigarettes, vehicles, incineration plant, hazardous waste landfill, and mines, as well as cement plant. We found a relationship between U-NAG and U-Hg and U-Cd in lower level exposure (above about 1 μg/g cr). According to studies using the Korean Nation Health and Nutrition Examination Survey (KNHANES), U-Hg and U-Cd were associated with decreased glomerular flow rate (GFR), despite low dose environmental exposure [24, 25, 32]. The result of our study also show a correlation between U-Hg and U-Cd and higher U-NAG. However, there was no correlation between B-Pb and higher U-NAG in our study. The relationship between blood lead levels and renal dysfunction at low-level environmental exposure in the general population is controversial.

Multiple exposure to higher heavy metal levels have high U-NAG compared to single exposure. Heavy metals may have an additive effect in renal toxicity. In previously published experimental studies in mice, concurrent exposure to arsenic and cadmium had more toxic renal effects than did single exposure [33, 34]. In a recent study, simultaneous exposure to cadmium and lead induced a reduction in GFR [25].

Diabetes and hypertension are major risk factors for chronic renal disease [35]. Diabetic glomerular sclerosis or hypertensive glomerular sclerosis induced slowly worsening albuminuria, hypertension, gradual decline of GFR, and nephrotic syndrome. Our results suggest that hypertension and diabetes were significantly related to higher U-NAG. The adjusted OR of higher U-NAG in hypertension was 1.67. The adjusted OR of higher U-NAG in diabetes is 2.51.

Air pollution exposure was divided into a higher exposure group in a rural area (MEG-R), a higher exposure group in a downtown area (MEG-D), and a lower exposure group (LEG). The adjusted OR of higher U-NAG in MEG-R and MEG-D compared to LEG was 2.07 and 4.07, respectively, in spite of the younger age population in MEG-D. We do not fully understand why the OR of higher U-NAG is large in MEG-D compared to that of MEG-R. We assumed it was because of wind direction, population density, and traffic exhaust.

In the group without heavy metal exposure, OR of higher U-NAG in MEG-R and MEG-D as compared to LEG was 2.13 and 4.79, respectively. Whereas in the group with heavy metal exposure (U-Cd or/and U-Hg), the OR of higher U-NAG in MEG-R and MEG-D compared with LEG was 6.49 and 8.12, respectively. This result suggests concurrent exposure to heavy metals and air pollution has additive toxic renal effects. Further, we assumed that the populations having higher heavy metal exposure have increased risk if they live in a high air pollution area.

We found an incomprehensible result, in that smoking was not correlated with U-NAG. In previous studies, smoking alters the proximal tubular function and leads to increased U-NAG by impairment of organic cation transport [36, 37]. Generally, smoking results in a higher concentration of toxins than does usual air pollution. Thus, no-correlation between smoking and U-NAG was not predicted. Further studies are needed to reveal additional information about smoking and U-NAG. The OR of U-NAG for manual work was high compared to that of non-manual work. We understood that manual work provides more opportunity for exposure than other causes during work. U-NAG as a nephrotoxic biomarker represents more recent damage than GFR. The GFR represent the cumulative effect of kidney damage by several risk factors such as diabetes, hypertension, glomerulonephritis, and many nephrotoxins [38].

The limitations of this study should be considered. First of all, the study design was cross sectional. It was insufficient to determine if there was a causal relationship. In the past, there was severe air pollution in the research region. However, an industrial dust collecting system was installed 20 years ago. After that, air pollution was greatly reduced. Nevertheless, the region around the cement plant has poor air quality because of cement dust from the plant and limestone dust from the strip mine. Accordingly, the results of this study might reflect cumulative air pollution exposure. On the other hand, we had to consider the underestimation effect due to early death and migration to other regions. Second, the lower level exposure group did not live in perfectly good air quality conditions. However, the people included in LEG lived 5 km from the cement plant and on higher ground surrounded by mountains. The result of PM10 and PM2.5 measured during 3 different seasons showed better air quality results than in the higher exposure area. Third, higher U-NAG does not directly mean chronic renal disease; it just indicates renal proximal tubular damage at that time. The results of our research merely suggest that air pollution can induce renal disease. The obvious conclusion is that further prospective study is needed to assess chronic renal disease or end stage renal disease, and mortality due to renal disease.

The main strength of this study is the evaluation of the relationship between air pollution and renal toxicity. We presented adjusted ORs by major risk factors for chronic renal disease, including diabetes, hypertension, smoking, and occupation. Further, we showed the result of a stratified analysis by heavy metals exposure and dose-response relationship.

## Conclusions

We presented the relationship between air pollution and higher U-NAG as a renal proximal tubular oxidative stress marker, through research conducted on residents living around a cement plant. The results were adjusted by major risk factors for chronic renal disease, including diabetes, hypertension, smoking, occupation, and heavy metals exposure. Especially, heavy metals exposure may have a synergic effect with air pollution in renal toxicity. Further study about the additional adverse effects of air pollution as a risk factor for kidney disease is needed.

## Abbreviation

B-Pb: Blood lead
DM: Diabetes mellitus
GFR: Glomerular flow rate
GM: Geometric mean
GSD: Geometric standard deviation
HbA1c: Hemoglobin A1c
HTN: Hypertension
LEG: Less exposed group
MEG-D: More exposed group from downtown area
MEG-R: More exposed group from the rural area
OR: Odds ratio
PM: Particulate matter
U-Cd: Urinary cadmium
U-Hg: Urinary mercury
U-NAG: Urinary n-acetyl-B-glucosaminidase
